# Single-cell insights into tumor microenvironment heterogeneity and plasticity: transforming precision therapy in gastrointestinal cancers

**DOI:** 10.1186/s13046-025-03567-5

**Published:** 2025-11-28

**Authors:** Jialei Weng, Feng Ju, Zicheng Lyu, Ningbo Fan, Daniel J. Smit, Wenxin Xu, Xiaolin Wu, Philip Becker, Yinan Xu, Michal R. Schweiger, Axel M. Hillmer, Ralf Harwig, Sheraz Gul, Alexander Link, Lydia Meder, Nan Fang, Qiongzhu Dong, Christiane J. Bruns, Ning Ren, Yue Zhao

**Affiliations:** 1 Department of General, Visceral, Thoracic and Transplantation Surgery, University Hospital of Cologne, Kerpener Straße 62, Cologne, 50937 Germany; 2 Department of Liver Surgery and Transplantation, Liver Cancer Institute, Zhongshan Hospital, Fudan University, Key Laboratory of Carcinogenesis and Cancer Invasion, Ministry of Education, Shanghai, 200032 P. R. China; 3 Key Laboratory of Whole-Period Monitoring and Precise Intervention of Digestive Cancer of Shanghai Municipal Health Commission, Shanghai, 201199 P. R. China; 4 Institute of Tumor Biology, University Medical Center Hamburg-Eppendorf, Hamburg, 20246 Germany; 5 Singleron Biotechnologies GmbH, Cologne, 51105 Germany; 6 Institute for Translational Epigenetics, Faculty of Medicine, University Hospital Cologne, Cologne, 50931 Germany; 7 Institute of Pathology, University of Cologne, Faculty of Medicine and University Hospital of Cologne, Cologne, 50937 Germany; 8 Center for Molecular Medicine Cologne, University of Cologne, Cologne, 50931 Germany; 9 Dep. Computational Molecular Biology, Max Planck Institute for Molecular Genetics, Berlin, 14195 Germany; 10 Fraunhofer Institute for Translational Medicine and Pharmacology ITMP, Hamburg, 22525 Germany; 11 Fraunhofer Cluster of Excellence for Immune-Mediated Diseases CIMD, Hamburg, 22525 Germany; 12 Department of Gastroenterology, Friedrich-Alexander University Erlangen-Nuernberg , Medical Campus Upper Franconia, Bayreuth, 95445 Germany; 13 Faculty of Medicine, Chair of Experimental Medicine 1, Friedrich-Alexander University Erlangen-Nuernberg, Erlangen, 91054 Germany

**Keywords:** Gastrointestinal cancers, Single-cell RNA sequencing, Heterogeneity, Plasticity, Tumor microenvironment, Cell subsets, Cancer-associated fibroblasts

## Abstract

The development and progression of gastrointestinal (GI) cancers not only depend on the malignancy of the tumor cells, but is also defined by the complex and adaptive nature of the tumor microenvironment (TME). The TME in GI cancers exhibits a complex internal structure, typically comprising cancer cells, cancer stem cells, cancer-associated fibroblasts, immune cells, and endothelial cells, all embedded within a dynamic extracellular matrix. This intricate ecosystem fuels tumor initiation, progression, metastasis, recurrence and therapy response through the heterogeneity and plasticity. Recent advances in single-cell sequencing have provided unprecedented resolution in profiling the cellular diversity and interactions within the TME. These technologies have uncovered previously unknown cell subtypes and intricate communication networks that drive therapy resistance and tumor relapse. In this review, we summarize and discuss the latest findings from single-cell sequencing of key cellular players and their interactions within the TME of GI cancers. We highlight single cell insights that are reshaping our understanding of tumor biology, with particular focus on their implications for overcoming therapy resistance and improving clinical outcomes. We believe that a deeper understanding of TME heterogeneity and plasticity at the single-cell level promises to transform the landscape of precision treatment in GI cancers.

## Introduction

With enhancements in methods to explore cancer tumor progression from the morphological level through to molecular genetics, our understanding of tumor biology has been revolutionized. It is now well recognized that even cancers of similar histologies can exhibit distinct phenotypes through different genomic as well as epigenetic aberrations and transcriptomic profiles leading to spatial and temporal variations within a tumor, with these being major determinants of the biological behavior and the clinical outcome of cancer disease [1]. When considering that the transcriptome profile of any single cell reflects very private traits of this cell, intercellular heterogeneity is therefore inevitable [2]. The recognition of the high heterogeneity, both, between and within tumors has given rise to the concept of individualized cancer medicine: cancer genomes should be deciphered at the individual level to precisely characterize disease biology and achieve therapeutic benefit through targeting certain critical and actionable genetic aberrations [3]. However, despite the growing number of cancer drugs that target specific genetic aberrations, our ability to evaluate the sensitivity and the response duration of an individual tumor, and the possibility to evolve drug resistance, remains unsatisfactory [4].

The advent of sequencing technologies has enabled the routine interrogation of the genomic landscapes of tumors, but so far only so called ‘bulk-sequencing’ has been used for diagnostic purposes which is the sequencing of tissues containing thousands of single cells [5]. However, this analysis, overlooks intercellular heterogeneity and fails to provide a comprehensive view of the tumor genetic landscape. To address this, single-cell sequencing (SCS) methods were developed, with the first single-cell DNA sequencing and first single-cell exome sequencing in human cancer cells performed in 2011 and 2012, respectively, with complicated intercellular heterogeneity being identified [6, 7]. Currently the most widely used SCS technology in oncology research is the single-cell RNA sequencing (scRNA-seq) [8]. An increasing number of studies integrate multiple omics data on single cells to collect more information and thereby provide more insightful disease profiles. For example, linking DNA and RNA profiles of individual cells by genome and transcriptome sequencing technologies can explain differences in transcript levels between cells in terms of genetic variations [9, 10]. Besides, single-cell spatial transcriptomics, which combines scRNA-seq with spatial transcriptomics, preserves the structures of tissues and interactions between cells and reveals the native sites of gene expression while retaining spatial information [11–13]. Collectively, the advent of various SCS technologies have greatly facilitated our identification of unknown cell types in tissues as well as our understanding of various cellular origins and cell lineage trajectories [14].

Gastrointestinal (GI) cancers, including esophageal, gastric, colorectal, liver, and pancreatic cancers, account for a quarter of all cancer cases and cause more than one-third of cancer-related deaths globally [15]. GI cancers are characterized by a wide variety of genetic mutations and microenvironmental reshaping [16]. These intricate pathogenetic mechanisms lead to a high heterogeneity and result in great variations in pathological types, treatment sensitivity, and clinical outcomes among individuals, which poses a great challenge for personalized systemic therapies of patients with GI cancers.

**Fig. 1:**
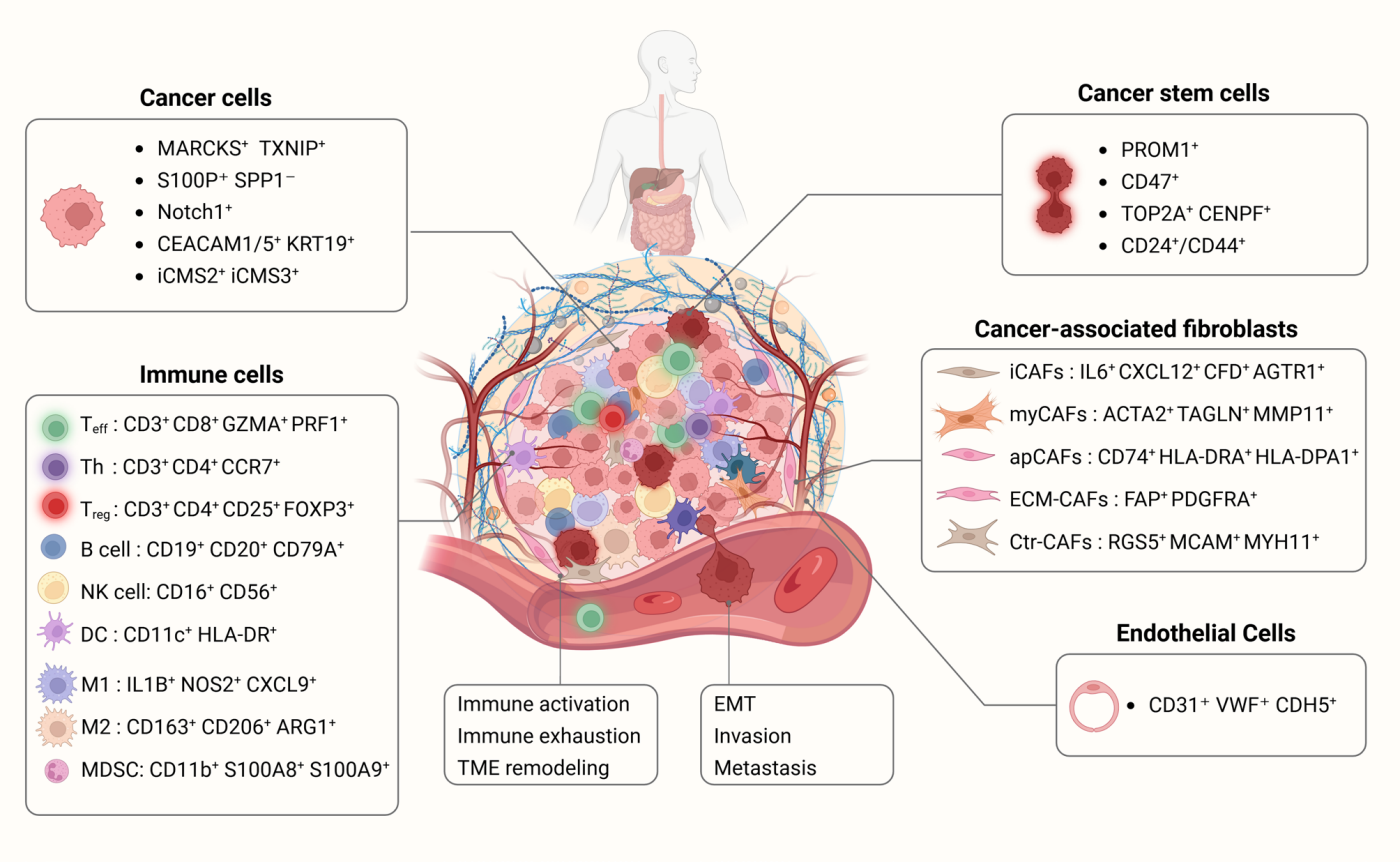
The heterogeneity of various cell types in the TME of GI cancers. TME in GI cancers has a complex internal structure, it usually includes cancer cells, CSCs, stromal cells, CAFs, immune cells, extracellular matrix, and extracellular vesicles. These components together contribute to the TME renewal and affect tumor plasticity. Different cell subsets show heterogeneity in different niches and play a critical role in tumor invasion, metastasis, and therapy resistance. In different GI cancers, specific cell clusters and molecular features have been identified by scRNA-seq. In gastric cancer, MARCKS⁺ and TXNIP⁺ high-plasticity clusters were detected, exhibiting a tendency to shift toward a highly proliferative phenotype. In ICC, S100P⁺ SPP1⁻ clusters were identified and associated with reduced immune infiltration. In ESCC organoids, knockout of Notch1 alone was sufficient to induce an immunosuppressive TME. In pancreatic cancer, CEACAM1/5⁺ and KRT19⁺ cells indicated poor prognosis. In CRC, iCMS2 is characterized by Wnt pathway activation resulting from APC mutations, whereas BRAF and KRAS mutations are enriched in iCMS3. CAF: Cancer-associated fibroblast; CRC: Colorectal cancer; CSC: Cancer stem cell; ESCC: Esophageal squamous cell carcinoma; GI: Gastrointestinal; ICC: Intrahepatic cholangiocarcinoma; MDSC: Myeloid-Derived Suppressor Cell; TME: Tumor microenvironment

Here, we aim to provide a single-cell insight into the heterogeneity and plasticity of the tumor microenvironment (TME) for GI cancers, with an emphasis on cell subsets (Fig. 1). In addition, this review will outline the key and actionable targets based on SCS in GI cancers which will offer the potential to revolutionize the treatment paradigms of GI cancers in the future.

###  Introduction to the scRNA-seq analysis process

**Fig. 2:**
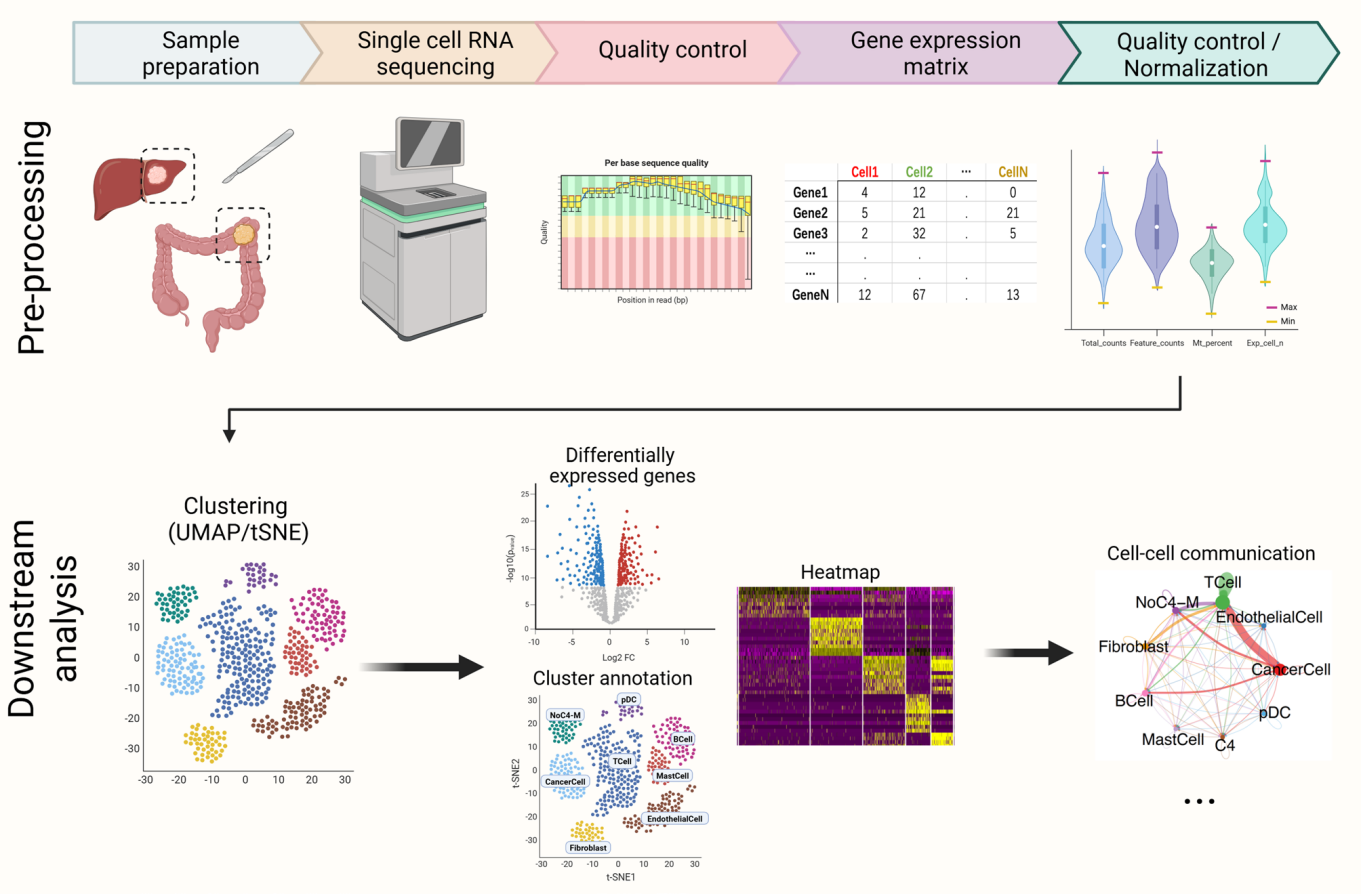
The single-cell RNA sequencing analysis workflow. After tissue samples are sequenced, the count matrices can be obtained. The raw data undergoes the pre‐processing and downstream analysis

scRNA-seq technology is rapidly evolving, with substantial progress made to improve its analytical outcomes. In this section, we first provide an overview of the technical issues related to scRNA-seq. ScRNA-seq data analysis can be subdivided in pre-processing and downstream analysis tasks [17] (Fig. 2). The first step in scRNA-seq is the high-quality capture of single cells from tissues, but the methods used to isolate individual cells from a tissue vary greatly among different tissues or cells. The most common single-cell isolation techniques include fluorescence-activated cell sorting and magnetic‐activated cell sorting [18]. From the scRNA-seq platform, raw sequencing data in the format of FASTQ or BCL can be acquired and be organized into read count matrices summarizing the expression of the different genes across the different cells [18]. For data preprocessing, the FASTQ raw data should pass through the first quality control pipeline, where FastQC is the most widely used software. In recent years, more software or platforms have been developed, such as Falco, HTSQualC, and NGSQC [19–21]. Typical pre-processing steps include data normalization, filtering out low quality cells or genes, batch correction, visualization components as well as potential feature selection, for example based on gene expression variability or biological functions. This analysis is usually performed on several major platforms including Seurat and Scran based on R language and Scanpy [22–24].

Downstream analysis tasks include dimension reduction and visualization such as principal component analysis (PCA), t-distributed stochastic neighbor embedding (t-SNE), and uniform manifold approximation and projection (UMAP) [25–27]. Graph-based cluster methods like K-nearest neighbor (KNN), then embed cells in graphs to cluster different principal components. In contrast to t-SNE, UMAP has gained wider adoption as it has advantages in reduced computational time for processing large-scale data and more optimized structuring of global datasets [27, 28]. Following clustering, during the cell type annotation step, cell subpopulations are annotated according to the respective marker gene or gene signature expression. Automatic annotation of cell types is preferred over manual annotation, which can otherwise be both time-consuming and subjective [29]. A study comparing 10 automatic annotation softwares, researchers determined Seurat as the best method for major type cell annotation due to its highest predictive robustness to complex data [30].

Other downstream analysis tasks include differential gene expression between cell subtypes or different experiments, gene enrichment analyses, trajectory inference (TI), and anylyses of inter-cellular communication. Enrichment analyses such as gene set enrichment analysis (GSEA), gene ontology (GO), and Kyoto Encyclopedia of Genes and Genomes (KEGG) analyses, can integrate the differential gene information into functional information with biological significance. TI aims to align cells based on their underlying dynamic processes according to pseudo time, which can be linear, nonlinear, or branching. It is becoming more popular in recent years, with more than a hundred tools or platforms currently developed [31]. Inter-cellular communication refer to the exchange of information between cells through a medium and then generate a corresponding response. Many databases and software have been built to record known ligand-receptor interactions to help analyze the cell communication, such as CellPhoneDB, CellTalkDB, and CellChat based on the transcript levels of ligands and receptors in scRNA-seq data [32–34]. In addition, a new computational method, called Numbat, has been shown to be able to combine haplotype information with alleles and expression signatures to enhance the detection of copy number variation in scRNA-seq, thus allowing for the reconstruction of tumor copy number profiles and the precise identification of genetic subpopulations of tumor cells with transcriptional signatures associated with tumor progression and therapeutic resistance [35]. Although it is increasingly recognized that mRNA can be regulated and only provides an indirect prediction of the resulting protein levels rather than a direct measure of expression, RNA-based single-cell sequencing remains a powerful tool for dissecting cellular heterogeneity and functional states in cancer research.

### Esophageal cancer

Esophageal cancer is the seventh most common cancer and the sixth leading cause of cancer-related deaths worldwide [15]. Esophageal squamous cell carcinoma (ESCC) and esophageal adenocarcinoma (EAC) are two major esophageal cancer subtypes [36]. In recent years, the utilization of SCS has substantially advanced our comprehension of esophageal cancer development and progression. Several studies have reported on the investigation of esophageal cancer initiation using scRNA-seq technology. By comparing ESCC and EAC tissues at the single cell level, distinct gene signatures, signaling pathways, and cancer stemness markers have been identified [37, 38]. Specifically, Notch signaling has been shown to be exclusively activated in ESCC [37]. Cell cycle-associated genes were inextricably associated with stemness in EAC, whereas DNA replication and DNA damage repair pathways have significant correlation with cell stemness in ESCC [38]. The analysis of SCS data in healthy human esophageal tissues and BE tissues has shown that BE originates from undifferentiated gastric cardia cells, and EAC likely arises from undifferentiated BE cells, paving the way for early detection strategies for EAC [39]. A mouse model replicating progression of human ESCC has shown a time-ordered single-cell transcriptomic analysis with five different precancerous and cancerous lesions in the esophagus [40]. This study led to the identification of distinct ESCC epithelial expression and transition signatures, providing a dynamic perspective on the oncogenic evolution of epithelial cells in ESCC. A complementary study analyzing five ESCC tissue samples led to the identification of a novel malignant cell subtype, termed confused cell identity and characterized by a mixture of molecular features from various normal squamous cell types [41]. Moreover, a more recent and in-depth scRNA-seq analysis of murine esophageal organoids has been conducted to investigate genetic factors influencing ESCC initiation and immune invasion [42]. The output of this work indicated that the triple-knockout of *Trp53*, *Cdkn2a*, and *Notch1* induces neoplastic characteristics of ESCC, while the knockout of *Notch1* alone leads to an immunosuppressive TME in ESCC (Table 1). Subsequent studies confirmed the Notch1 subtype in ESCC patients, suggesting that *Notch1* mutation may serve as a predictive biomarker for longer overall survival (OS) with tislelizumab compared to chemotherapy, potentially associated with enrichment of IFN-I signatures and reduced infiltration of B cells and neutrophils [55].

**Table 1:** Cancer cell subsets and their involved pathways/functions in GI cancers

Cancer type	Subset	Sample size	Pathway	Characteristic/Function		Ref	
Esophageal cancer	TPM4^+^	5	JAK/STAT-SOX2	Promote the aggressiveness		[41]	
	PCN-type	4 + 69	CCL2–CCR2 axis	Induces esophageal neoplasia and immune evasion		[42]	
Gastric cancer	GA-FG-CCP	12	Wnt/β-catenin	-		[43]	
	EBV^+^	12	Interferon-α response, antigen processing and presentation	-			
	C2	35	-	High autophagy, high plasticity		[44]	
	C3	35	-	High mTORC1 activity, high proliferation			
Colorectal cancer	stem/TA-like, goblet cell-like, and TC1-4	12	Wnt, YAP and MAPK	Cancer stemness		[45]	
	E/133^+^ 44^+^, E/133^+^, E/44^+^	1	-	Cancer stemness		[46]	
	iCMS2	63	Wnt, MAPK	inactivating APC mutations		[47]	
	iCMS3	63	Wnt, MAPK, TGF-β	more frequent KRAS, PIK3CA and BRAF mutations			
Liver cancer	PROM1^+^/CD47^+^	7	EMT activation	TME remodeling and tumor metastasis		[48]	
	TOP2A^+^/CENPF^+^	3	TOP2A/β-catenin/YAP1	promoted HCC stemness and overgrowth		[49]	
	CD24^+^/CD44^+^	1	Lipid metabolism	self-renewal		[50]	
	S100P^+^SPP1^−^, S100P^−^ SPP1^+^	28	-	optimal biomarkers for iCCA^phl^ and iCCA^pps^		[51]	
Pancreatic cancer	Type 2	35	PI3K, Hedgehog, Wnt and Notch	Poor prognosis		[52]	
	EMT^+^	16/5	EMT	Poor prognosis	[53, 54]		

In order to delve into the TME of esophageal cancer, the plasticity and heterogeneity of esophageal cancer tissues at the single-cell resolution has been investigated. The scRNA-seq of 60 ESCC tissues led to the identification of 8 common expression programs from ESCC tumor cells, 26 immune cell and 16 stromal cell subtypes, offering a comprehensive overview over the composition of the ESCC microenvironment [56]. More importantly, the investigators found that the TME of ESCC is in an immunosuppressive state, characterized by the high infiltrations of exhausted T cells, regulatory T cells (Tregs) and myeloid cells. In addition, they revealed the role of two intermediate phenotypes of cancer-associated fibroblasts (CAF1 and CAF2) as well as multiple cellular interactions such as the crosstalk between Th17 cells and CAF2 in the progression of ESCC. Another study analyzing the scRNA-seq data of five ESCC tumors unveiled enhanced hypoxia and oxidative phosphorylation in ESCC cells compared to normal esophageal cells, with this metabolic reprogramming driving proliferation, metastasis, and drug resistance [57]. Further analyses revealed that multiple stromal cell, B cell and macrophage phenotypes were present in the ESCC and primarily promoted tumor progression, in contrast, T cell subclusters displayed anti-tumor immune responses, but CD8^+^ T cells showed great heterogeneity and tended to be exhausted in ESCC, which is consistent with previous studies [58]. In addition, the co-analysis of scRNA-seq and microarray-based spatial transcriptomics in three ESCC tissues, unveiled the spatial distribution of different cell subsets within the TME as well as the potential crosstalk among cells [59]. The researchers found that the crosstalk between epithelial cells and fibroblasts was the most significant and tumor cells exhibited strong inhibitory interactions with natural killer (NK) and T cells such as TIGIT-NECTIN2. A comprehensive study involving 79 multi-stage esophageal lesions, highlighted the crucial role of ANXA1 and its receptor FPR2 in mediating crosstalk between epithelial cells and fibroblasts in promoting ESCC [60]. In this study, a gradual and significant reduction in ANXA1 expression within epithelial cells, leading to the transformation of normal fibroblasts into CAFs was observed. Moreover, CST1^+^ myofibroblasts as a tumor-specific fibroblast subtype, which is associated with poor prognosis in ESCC were identified [61] (Table 2). These scRNA-seq data were further integrated with gene microarray profiles from 84 ESCC tumors samples, which identified CCL18 as a promising therapeutic target for impeding ESCC progression [73]. Similarly, a comprehensive immune suppressive landscape in the ESCC TME based on the scRNA-seq of CD45^+^ cells has been described [74]. This landscape was marked by the dominance of exhausted T and NK cells, Tregs, as well as M2 macrophages and tolerogenic dendritic cells (DCs). Furthermore, analysis of tumor tissues, normal tissues, normal and metastasis lymph nodes at single cell levels and revealed that the development of interferon-induced T/B cells, POSTN^+^ myofibroblasts, and APOC1^+^APOE^+^ macrophages, along with the inter-cellular interactions, collectively contributes to the establishment of a premetastatic niche and the facilitation of metastasis within ESCC [75].

**Table 2:** CAFs subsets and their involved pathways/functions in GI cancers

Cancer type	Subset	Sample size	Pathway	Characteristic/Function	Ref
Esophageal cancer	CST1^+^ myofibroblasts	11	ECM, EMT, protein secretion and TGF-β	Poor prognosis	[61]
Gastric cancer	eCAFs	8	-	Promote metastasis/Poor prognosis	[62]
	iCAFs	8	-	communication with immune cells	
	CTHRC1^+^	9	ECM remodeling	Poor prognosis	[63]
	COL14A1^+^	9	-	high expression of C7 and APOD	
	MMP1^+^/MMP3^+^/MMP9^+^	9	-	high expression of TWIST1	
	STF3	31	TGF-β (activin–inhibin signaling module)	Poor prognosis	[64]
Colorectal cancer	CAF-A	11 + 7	TGF-β, ECM remodeling	High expression of MMP2, DCN and COL1A2	[65]
	CAF-B	11 + 7	TGF-β	high expression of ACTA2, TAGLN and PDGFA	
	mCAFs	56	Paracrine signaling	Affect angiogenesis and wound healing	[66]
	iCAF	56	EMT	Shape the immunosuppressive microenvironment	
	cS28,29	62	ECM remodeling	Enriched around dilated blood vessels at the colonic luminal margin	[67]
	F2_MCAM	6	NOTCH	Enriched with JAG1 and NOTCH3	[68]
	F4_F3	6	Complement and inflammatory response pathway	enriched with C3 and CXCL1, poor prognosis	
Liver cancer	iCAF	24	HGF-MET	Promote ICC growth	[69]
	myCAF	24	Has2	Promote tumor growth	
	vCAF	7	Vascular smooth muscle contraction response to calcium ions	Characterized by signature microvasculature genes	[70]
	mCAFs	7	EMT	Express low levels of α-SMA and high levels of ECM signatures	
	lpmCAFs	7	ECM, ROS, lipid metabolism	Express high levels of lipid metabolism-related genes	
	lpCAFs	7	protein-lipid complex remodeling	Expressed high levels of lipid processing markers	
	apCAFs	7	MHC-class-II protein complex, antigen processing and presentation	Expressed major histocompatibility complex II (MHCII) genes and chemokine-related genes	
	ECM-CAF	5	ECM remodeling, collagen production, Wnt/β-catenin	Promote cancer cell growth and angiogenesis	[71]
	Ctr-CAFs	5	-	Expressed blood vessel wall markers and contractility marker	
Pancreatic cancer	myCAF	6	TGFβ/SMAD2/3	αSMA^high^ IL-6^low^ nonneoplastic origin	[72]
	iCAF	6	JAK/STAT	αSMA^low^ IL-6^high^	
	apCAFs	6	Antigen presentation and processing, fatty acid metabolism, MYC targets and MTORC1 signaling	Expresse MHC class II-related genes	

The primary clinical management for esophageal cancer patients includes multimodal interdisciplinary treatments, including surgery, chemotherapy, radiotherapy, targeted therapy, and immunotherapy [76]. Thus, studies have employed scRNA-seq into the investigation of therapy response in esophageal cancer. For example, mapping of the distribution of N6-methyladenosine (m6A) methylation at the single-cell level led to the development of a model for predicting prognostic risk, immune infiltration, and chemotherapy sensitivity in ESCC [77]. A study further compared pre- and post-neoadjuvant chemoradiotherapy (NCRT) ESCC samples, revealing comprehensive NCRT-induced immune changes in the TME and these findings offer novel insights into the immunological mechanisms underlying ESCC patients response to NCRT [78]. Similarly, the functional changes of tumor-infiltrating B lymphocytes (TIL-Bs) in ESCC patients’ tissues with and without chemotherapy using scRNA-seq, providing a novel perspective on the heterogeneity of TIL-Bs in ESCC patients during the chemotherapy have been investigated [79]. Besides, scRNA-seq analysis conducted on ESCC patients undergoing neoadjuvant chemoimmunotherapy, revealed that responders exhibit a higher proportion of CD8^+^ effector memory T cells but a lower proportion of CD4^+^ Tregs in TME [80]. In addition, researchers sorted T cells from 8 ESCC patients and identified 12 subclusters within CD8^+^ T cells and 4 subclusters within CD4^+^ T cells using scRNA-seq [81]. Their analysis unveiled a distinctive link between sprouty RTK signaling antagonist 1 (SPRY1) expression and exhausted CD8^+^ T cells, suggesting a potential therapeutic target for reversing the immunosuppressive TME in ESCC. Analysis of peripheral blood mononuclear cells (PBMCs) from both PD-1 antibody-sensitive and resistant ESCC patients, led to the successful identification of important pathways (like PD-L1 and PD-1 checkpoint pathways and B-cell receptor signaling pathways) and gene signatures associated with immunotherapy resistance of ESCC patients [82]. Moreover, bulk RNA-seq led to the identification of upregulated gene signatures in EAC patients responding to immunochemotherapy, which were subsequently confirmed to be specifically expressed in T cells and NK cells through scRNA-seq [83]. Additionally, scRNA-seq results highlighted that EAC patients with high tumor monocyte content exhibited increased sensitivity and survival benefits from the therapy. In addition to utilizing scRNA-seq in tumor tissues, paclitaxel-resistant ESCC cell lines were established and compared to resistant cells to control cells using both bulk RNA-seq and scRNA-seq, which uncovered both intrinsic and acquired paclitaxel resistance signatures in ESCC, offering insights into potential targeting strategy for resistant ESCC patients [84].

In summary, the application of SCS technologies has substantially expanded our understanding of various facets of esophageal cancer, encompassing tumor initiation, TME, and treatment responses. By analyzing individual cells, scRNA-seq has offered valuable insights into the intricate biology of esophageal cancer, holding the potential for the development of more precise diagnostic and therapeutic approaches in the future.

### Gastric cancer

Gastric cancer is the fifth most common cancer, causing one in 13 cancer deaths worldwide in 2020, with a five-year survival rate of around 25% [15]. Depending on the tumor stage, treatment strategies for gastric cancer are categorised as curative or palliative, and the traditional palliative regimen is based on cytotoxic chemotherapy with targeted therapy and immunotherapy as adjuncts [85]. In terms of targeted therapies, anti-HER2 (trastuzumab) and anti-VEGFR (ramucirumab) antibodies have been approved for use in combination with chemotherapy for gastric cancer [86, 87]. Immunotherapy, on the other hand, has shown clinical efficacy against gastric cancer in several clinical trials, particularly in patients with mismatch repair-deficient (dMMR) or high CPS scores [88, 89]. However, patients with gastric cancer still suffer from limited therapeutic efficacy of these emerging treatments. With the deepening of our basic scientific understanding of gastric cancer, the extremely diverse pathogeneses and oncogenic pathways of gastric cancer as a heterogeneous disease have been gradually recognized as an important reason for the poor treatment responses in gastric cancer.

The heterogeneity of gastric cancer has been revealed at single-cell resolution: single-cell whole-exome sequencing of one gastric adenocarcinoma patient identified 117 non-synonymous somatic mutations in tumor cells [90]. Importantly, two genes that may play a role in promoting carcinogenesis, namely cell division cycle 27 (*CDC27*) and filaggrin (*FLG*), were found to be mutated frequently at both single-cell and population levels. Subsequently, in 2021, a scRNA-seq study in nine gastric adenocarcinomas identified five tumor cell subsets with distinct transcriptional profiles and revealed a high diversity of differentiation degrees within and between tumors [43] (Table 1).

SCS studies based on trajectory analysis and functional enrichment analyses also play an important role in decoding the underlying mechanisms contributing to gastric cancer metastasis. ScRNA-seq of metastatic lymph nodes of gastric cancer revealed dynamic genetic alterations in tumor cells during metastasis to lymph nodes and identified a tumor cell subtype with potentials of lymph node metastasis [91]. Besides, it has been found that the suppression of neutrophil polarization-related genes and the activation of the immune checkpoint SPP1 could promote lymphatic metastasis in gastric cancer. As peritoneal metastasis is very common in gastric cancer, intra-tumoral heterogeneity and lineage diversity of peritoneal metastases from gastric cancer were also observed at the single-cell level [44, 92]. Evolutionary trajectory analysis further identified high-plasticity gastric cancer clusters with a tendency to shift to a highly proliferative phenotype, which could be marked by two autophagy-related genes (*MARCKS* and *TXNIP*) and targeted by autophagy inhibitors. These findings provide insights into the developmental trajectory of tumor cells underlying gastric cancer progression.

SCS analysis has also been applied to tumor stroma to provide deeper insights into the TME in gastric cancer. Several scRNA-seq-based studies have found that gastric cancer tissues are significantly enriched for stromal cells, macrophages, DCs, and Tregs, with an increased proportion of plasma cells as a novel feature of diffuse-type tumors [63, 64, 93]. In addition, both immune cells and stromal cells exhibit significant cellular heterogeneity, and receptor-ligand analyses further revealed the unique inter-cellular communications in the gastric cancer TME [93, 94]. For example, gastric cancer cells and many immune cell subtypes can interact with cytotoxic/exhausted CD8^+^ T cells and/or NK cells via HLA-E-KLRC1/KLRC2, suggesting that the anti-KLRC1 antibody may be a potential therapeutic option in gastric cancer [94]. Single-cell studies have provided insights into the molecular characterization of the gastric cancer TME during disease progression and therapy. Analyzing precancerous, localized, and metastatic gastric adenocarcinoma (GAC) six TME ecotypes were identified (EC1–6), whereas EC4, EC5, and EC2 are enriched in normal tissues, precancerous lesions, and metastatic tumors, respectively, while EC3 and EC6 represent distinct primary GAC ecotypes correlating with histopathology, genomic features, and survival outcomes [95]. In addition, the researchers found that extensive stromal reshaping occurs during GAC progression. During peritoneal metastasis of gastric cancer, an increased proportion of monocyte-like DCs, which are pro-angiogenic and have a compromised antigen-presenting capacity, was observed [44]. Heterogeneity and remodeling of the TME are also important restraints of gastric cancer response to chemotherapy [96]. In addition, an absence of separate exhausted CD8^+^ T cell clusters and low expression levels of exhaustion markers in gastric cancer tissues, which is considered as evidence for the poor efficacy of immunotherapy in gastric cancer have been reported [63]. Besides, PD-1 levels in CD8^+^ T cells may be able to be used to predict the clinical response of gastric cancer to PD-1 blockade therapy [94]. Moreover, a recent study based on scRNA-seq and paired scTCR/BCR-seq showed that antigen-specific ISG-15^+^CD8^+^ T cells are able to predict immunotherapy responses in Epstein-Barr virus (EBV)-associated gastric cancer patients [97]. The ISG-15^+^CD8^+^ T cell phenotype that reemerges after treatment could generate a CXCL13-expressing effector T cell subtype in responsive tumors but enters a terminal exhaustion state in non-responsive tumors in a LAG-3-dependent way. Thus, anti-LAG-3 therapy may effectively reverse drug resistance in patients with refractory EBV (+) gastric cancer.

In gastric cancer, CAFs are one of the key components within TME that promote or prevent tumorigenesis [98]. However, the heterogeneity and underlying mechanisms of CAFs in gastric cancer remain unclear, slowing down the translational progress of targeting CAFs (Table 2). A study of four subsets of CAFs with different properties, albeit all promoting tumors have been reported [62]. Meanwhile, they revealed the interaction of these CAFs with surrounding immune cells to construct a tumor-favorable TME. Another study found a tight interaction between CAFs and endothelial cells in gastric cancer, suggesting an important role in tumor angiogenesis [63]. In addition, a CAF subset with high expression levels of INHBA and FAP might be a predictor of poor clinical outcomes in gastric cancer [64]. However, despite the recognition of the heterogeneity and importance of CAFs in gastric cancer, the progress of functional studies is still relatively slow as the isolation and enrichment of specific CAFs remains a great challenge.

Overall, studies at the single-cell level suggest that gastric cancer tissue has an immunosuppressive TME. It has been hypothesised that *H. pylori* infection, one of the most common triggers of gastric carcinogenesis, may play an important role in fine-tuning the TME in gastric cancer. In addition to providing an opportunity to understand the heterogeneity of gastric cancer at the molecular level, they reveal complex patterns of cell-cell interactions and the relevance of some immune features to clinical outcomes, which will be a valuable resource for the development of novel targeted therapies for gastric cancer.

### Colorectal cancer (CRC)

CRC accounts for 10% of cancer cases and deaths worldwide, ranking third in incidence and second in mortality in 2020 [15]. Surgery can cure early-stage CRC, but outcomes worsen in advanced disease, highlighting the need for new biomarkers and therapeutic targets [99]. Most CRC arise from adenomas driven by *APC* mutations or serrated polyps with *BRAF* mutations. Around 15% of CRC display microsatellite instability (MSI) caused by epigenetic silencing or mutation of mismatch repair genes [100]. Unlike mismatch repair-proficient CRC, dMMR and MSI high CRCs, specifically patients with Lynch-Syndrome, are responsive to the immune checkpoint inhibitors.

A recognized feature of CRC is the existence of a small population of cancer stem cells (CSCs), which are implicated in tumor growth, metastasis, and therapy resistance [101]. Both snRNA-seq and scATAC-seq have been used to investigate the gene expression as well as chromatin accessibility in CRC and both have shown that both pre-cancerous polyps and CRCs are enriched in CSCs and immature epithelial cells compared to normal samples [102]. Over the course of cancer progression, these CSCs also adopt an increasing number of characteristics not observed in normal stem-cells such as changes in accessibility of certain transcription factor motifs, resulting in an increase in Wnt signaling along a proposed “malignancy continuum”. In contrast, stem-like properties have been shown to be adopted by CRC cells along a decreasing MAPK activity gradient independent of Wnt [45] (Table 1). However, these discrepancies could be explained by the examination of different CRC developmental stages in these studies. Additionally, there is evidence for “bidirectional conversion” during which CRC cells de-differentiate and regain stem-like properties [103]. In CSCs, mutational processes are active which do not occur in normal stem cells and drive mutational diversification or clonal evolution to increase heterogeneity of CSCs [46]. This intra-tumoral heterogeneity presents challenges for both CRC diagnosis and treatment.

In CRC, four consensus molecular subtypes (CMS1-4) were proposed by the CRC Subtyping Consortium (CRCSC) based on the bulk RNA sequencing data from over 4,000 patients [104]. Recent multi-omics studies have refined CRC subtype–etiology links; scRNA-seq of tumor cells for CMS classification revealed widespread intra-tumoral heterogeneity and shared features between subtypes [105]. However, while hybrid phenotypes at single cell level were identified, most cells can be assigned to a single subtype. Other scRNA-seq studies in CRC corroborate this conclusion such as the study of scRNA-seq data of 50,000 epithelial cells from 63 CRC patients that led to two malignant epithelial cell subtypes, termed iCMS2 and iCMS3 which can be used as a refinement of the CRCSC classification [47]. iCMS2 is characterized by Wnt pathway activation as a results of *APC* mutations while *BRAF* and *KRAS* mutations are enriched in iCMS3. Interestingly, it has been proposed that iCMS2 likely originates from crypt bottom stem cells, whereas iCMS3 may arise from serrated polyps. Moreover, crypt bottom genes are upregulated in iCMS2 and genes related to gastric metaplasia are upregulated in iCMS3. Linking these findings with those of scRNA-seq datasets from 29 adenomas and 19 serrated polyps and identified distinct cell origins [106]. While conventional adenomas may derive from epithelial stem cells residing at the bottom of intestinal crypts, serrated polyps develop from differentiated cells at the luminal surface through metaplasia.

The TME also plays an important role in tumorigenesis, metastasis and treatment response of CRC [107]. Using parallel single-cell genome and transcriptome sequencing, increased somatic copy number alterations in fibroblasts within TME have been shown in CRC compared to healthy tissues [108]. Remarkably, fibroblasts collected from different tumors were enriched in somatic trisomy of chromosome 7, suggesting selective pressure is driving expansion of genetically abnormal fibroblast in the TME. This was further extended by the identification of two distinct populations of CAFs, termed CAF-A and CAF-B, in CRC [65] (Table 2). CAF-A cells express genes with functions in extracellular matrix remodeling while CAF-B cells express markers of activated myofibroblasts. A study of 6 common cancer types leveraging integrated analysis of spatial and single-cell transcriptomics of over 740 K single cells, led to the identification of four CAFs subgroups [66]. Two subgroups, matrix CAFs (mCAFs) enriched for expression of genes related to angiogenesis and wound healing, and inflammatory CAFs (iCAFs), which appear to be involved in creating an immunosuppressive TME, were found in all cancers included in the study. Notably, spatial transcriptomic analysis showed that mCAFs and iCAFs populate distinct locations within the TME and there is evidence that CAFs can transition between the two states, highlighting the importance of interactions and cellular plasticity within the TME. Spatial and single-cell transcriptomics on microsatellite stable and MSI tumors have identified 11 fibroblast subsets including two separate iCAFs subsets and these iCAFs are enriched at the luminal tumor surface and create an inflammatory hub, which promotes tumor growth [67]. An anti-tumor immunity hub enriched in activated CXCL13^+^ T cells was identified only in MSI tumors and the presence of these T cells in MSI tumors has been proposed to provide an explanation for their susceptibility to immune checkpoint inhibitor treatment [109]. In addition, CAFs examined in both primary and metastatic CRCs led to the identification of distinct transcriptional programs such that MCAM^+^ fibroblasts are more prevalent in liver metastases and might play a role in facilitating the generation of CXCL13^+^ T cells, and associated with improved clinical outcomes [68]. Conversely, CD142^+^ fibroblasts are enriched in primary tumors where they promote invasiveness and angiogenesis through secretion of pro-tumoral factors. Likewise, using single-cell and spatial transcriptomics, differences in the TME of primary and metastatic tumors, with a strong enrichment of immunosuppressive cells in the latter have been uncovered [110]. These differences also correlated with response to neoadjuvant chemotherapy, with responsive tumors exhibiting higher CD8^+^ T cell infiltration and unresponsive tumors enriched in immunosuppressive macrophages, suggesting potential strategies to improve therapeutic outcomes.

Single-cell and spatial transcriptomics also enable the elucidation of interactions between cells in TME of CRC. For example, two distinct tumor-associated macrophages (TAMs) populations, namely C1QC^+^ and SPP1^+^ TAMs, with only the latter being specifically enriched in CRC have been identified [111]. SPP1^+^ TAMs were shown to interact with FAP^+^ fibroblasts to cooperate in the formation of an immune-exclusive, desmoplastic TME in CRC [112]. Additionally, undifferentiated HLA-G^+^ tumor cells may contribute to the polarization of macrophages to an SPP1^+^ phenotype, further inducing an immunosuppressive TME [113]. Digital spatial profiling of early-stage CRC also revealed immune modulation by cancer cells which was accompanied by increased infiltration of immunosuppressive TAMs [114]. Together, these studies emphasize how tumor cells establish a self-sustaining and self-promoting TME with complex interactions.

### Liver cancer

Liver cancer, which ranked sixth in incidence and third in mortality worldwide in 2020 [15], comprises three histologic subtypes: hepatocellular carcinoma (HCC), intrahepatic cholangiocarcinoma (ICC), and combined hepatocellular and intrahepatic cholangiocarcinoma (CHC) [115]. Although VEGF-targeted therapy combined with immunotherapy has expanded treatment options for advanced HCC, its effectiveness remains limited, especially in patients with esophageal and gastric varices due to portal hypertension [116].

Intra-tumoral molecular heterogeneity in HCC has been found to be partially attributable to the presence of CSCs, which are phenotypically and functionally heterogeneous [117]. A spatial transcriptomic study in liver cancer revealed that PROM1^+^ and CD47^+^ CSCs niches are associated with TME remodeling and tumor metastasis [48] (Table 1). In addition, another study based on scRNA-seq analysis identified a new CSCs subtype with positive expressions of TOP2A and CENPF, which could be regulated by M2-like macrophages and promoted HCC stemness and overgrowth [49]. Single-cell transcriptome sequencing also identified a CD24^+^/CD44^+^ CSCs subpopulation, which may be responsible for self-renewal of HCC cells [50]. Overall, single-cell genomics has successfully uncovered several previously undiscovered subtypes of CSCs with key functions in liver cancer, facilitating the development of therapy strategies targeting CSCs.

**Fig. 3:**
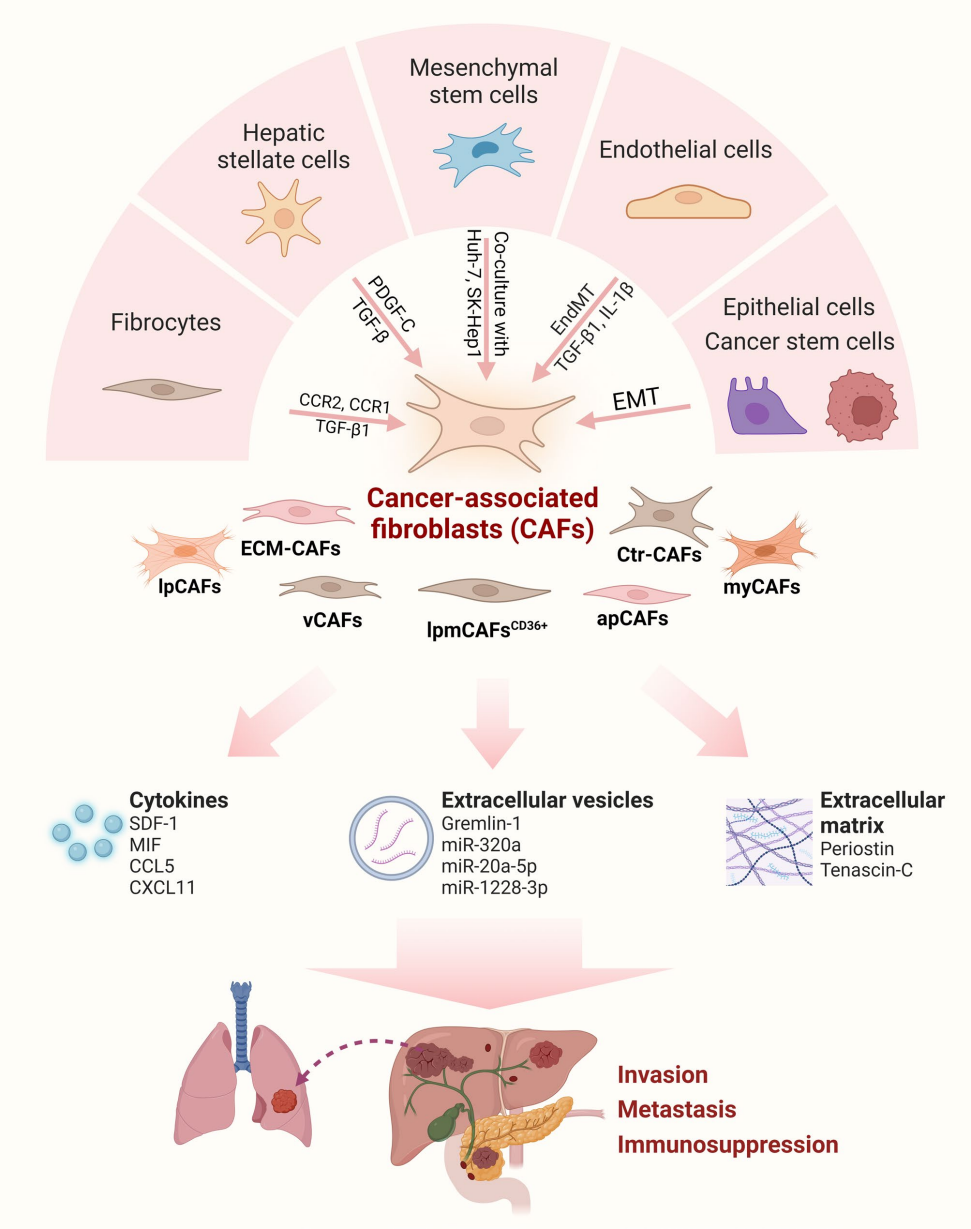
The origin and function of CAFs in liver cancer. CAFs in liver cancer can be derived from a variety of cells, including HSCs, mesenchymal stem cells, endothelial cells, fibrocytes, epithelial cells and CSCs. CAFs in the TME can secrete cytokines, extracellular vesicles, and extracellular matrix to regulate the functions of other cells, such as immune cells and cancer cells, which ultimately affect the tumor invasion, metastasis, and immunosuppression. CAF: Cancer-associated fibroblast; CSC: Cancer stem cell; GI: Gastrointestinal; TME: Tumor microenvironment

CAFs are one of the most abundant and versatile components within tumor stroma in liver cancer. Numerous studies have demonstrated that CAFs can be derived from a diverse range of cell types, including hepatic stellate cells (HSCs), mesenchymal stem cells, endothelial cells, epithelial cells, fibrocytes, and even CSCs [118] (Fig. 3). HSCs can be converted into myofibroblast-like cells by hepatocyte-derived PDGF-C [119]. Mesenchymal stem cells can also be transformed into CAFs-like cells after co-culturing with HCC cells [120]. In addition, endothelial cells, epithelial cells, and CSCs are also able to be an origin of CAFs through endothelial to mesenchymal transition or epithelial-mesenchymal transition (EMT) [121, 122]. In the TME of liver cancer, CAFs can release cytokines, extracellular vesicles, and extracellular matrix, which promote tumor migration, metastasis, and immunosuppression [123]. Some studies based on scRNA-seq have classified CAFs into iCAFs and myofibroblastic (myCAFs) subsets in liver cancer and highlighted the critical role of interactions between these CAF subtypes and tumor cells in disease progression [69, 124] (Table 2). Furthermore, five subtypes of CAF (vCAFs, mCAFs, lpmCAFs, lpCAFs, and apCAFs) have been identified in HCC [70]. Notably, this reported that lpmCAFs-derived migration inhibitory factors promote the recruitment of CD33^+^ myeloid-derived suppressor cells (MDSCs). Targeting lpmCAFs using CD36 inhibitors could restore the anti-tumor T-cell responses and synergize with immunotherapy in HCC. In addition, CAFs with different phenotypic characteristics, termed ECM- and Ctr-CAFs, are present in colorectal cancer liver metastases (CRC-LM) [68, 71]. Genes related to angiogenesis and ECM remodeling are highly expressed in ECM-CAFs, while Ctr-CAFs show greater contractility. ECM-CAFs arise from portal fibroblasts (PFs) expressing fibrillating collagens, whereas Ctr-CAF-I and Ctr-CAF-II derive from vascular smooth muscle cells and HSCs, respectively. PF-derived CAFs are abundant in ICC and CRC-LM, while HCC mainly contains HSC-derived CAFs. Notably, the ECM-CAF-specific protein LTBP2 can be targeted by antibodies in *vitro*, offering a way to remodel ECM-CAF-rich TMEs in ICC and CRC-LM [125]. Although depletion of CAF may not be sufficient to eradicate the tumor, this intervention may be used in combination with other therapies such as anti-angiogenic compounds to realize the synergistic effect [126].

Another important component within the complex ecosystem of liver cancer is the variety of immune cells, whose function status profoundly influence the anti-tumor effects of immunotherapy. However, traditional approaches such as immunohistochemistry and flow cytometry provide limited insights into the TME features of liver cancer. In a single-cell sequencing analysis, liver cancer was categorized into five TME subtypes, including immune activation, immune suppression mediated by myeloid or stromal cells, immune exclusion, and immune residence phenotypes [127]. Notably, researchers found that tumor-associated neutrophils are enriched in the myeloid cell subtype, which is associated with poor prognosis, and *CCL4*,* PD-L1*, and *SPP1* are promising targets for modulating this subtype. In another study, liver cancers were classified into five different TME subtypes, with patients characterized by advanced fatigue or rare T cell inflammation suffering a worse prognosis [128]. In addition, researchers found that SLC2A1 promotes the formation of immunosuppressive liver metastases by increasing the proportion of SPP1^+^ macrophages. In a comprehensive study integrating single-cell and bulk-tissue sequencing data, researchers found that Treg cells in HCC uniquely overexpress *TNFRSF4*,* TIGIT*, and *CTLA4* and are enriched for glycolysis/gluconeogenesis pathways [129]. The heterogeneity of NK cells and memory B cells was also observed in HCC. Interestingly, the immune ecosystem in the early recurrent HCC is characterized by a decreased proportion of immunosuppressive Treg cells and an increased proportion of DCs and innate-like CD161^+^ CD8^+^ T cells with dysfunctional cytotoxicity and low expansion [130, 131]. Meanwhile, expression of immune checkpoint genes such as *CTLA4*, *HAVCR2*, and *TIGIT* are reduced in T cells from recurrent tumors compared to the primary tumors, suggesting that checkpoint blockade approaches may be effective in primary HCC but not in recurrent tumors. In contrast, recurrent tumor cells show increased *CD47* expression. These results suggest that different therapeutic approaches are needed to treat primary or recurrent HCC, that anti-CD47-based therapies may be a viable preventive option for early recurrence of HCC. Besides, deep scRNA-seq on T cells found that exhausted CD8^+^ T cells and Tregs are preferentially enriched in HCC [132]. For macrophages, two subtypes with opposite clinical relevance have been identified in CRC-LM, one subtype expressing SERPINB2 with inflammatory features and positively associated with relapse-free survival, whereas the other subtype expressing *GPNMB*, defined as TAMs, is associated with poor prognosis [133]. In addition to providing systematic insights into the global TME of liver cancer, SCS can find unrecognized immune cell subtypes and their key characteristics in adaptive and intrinsic immunity. For example, FOXP3^+^ CXCR3^+^ innate-like mucosa-associated invariant T cells have been shown to be a subset with immunosuppressive potential using the scRNA-seq and flow cytometry analyses [134]. Another study emphasized the important role of LAMP3^+^ DCs clusters in lymph node metastasis in liver cancer [135].

SCS studies are also increasing in ICC. Recently, the World Health Organization and European Network for the Study of Cholangiocarcinoma have recognized that ICC can be classified into two main histological subtypes, including perihilar large duct type (ICCphl) and peripheral small duct type (ICCpps) [136]. To this end, S100P and SPP1 have been shown to be two markers for ICCphl and ICCpps, respectively [51]. S100P^+^ SPP1^−^ ICCphl has significantly reduced levels of infiltrating CD4^+^ T cells, NK cells, and increased proportions of CCL18^+^ macrophages and PD-1^+^ CD8^+^ T cells compared to S100P^−^SPP1^+^ ICCpps. In addition, Xiang et al. reported that ICC has significantly higher degrees of intra-tumoral heterogeneity than HCC [137]. The TME of ICC could be classified into four immune subtypes, including subtype I1 characterized by immune desert, subtype I1 by immunogenic reaction, subtype I3 by myeloid enrichment, and subtype I4 by mesenchymal features [138]. Furthermore, three immune subgroups with distinct clinical, genetic, and molecular features, IG1 (immunosuppression), IG2 (immune exclusion), and IG3 (immune activation) have been identified [139]. IG1 shows over-infiltration of neutrophils and immature DCs, IG2 is marked by high tumor proliferation and purity, and IG3 is enriched in adaptive immune cells, NK cells, and activated DCs. IG1 may respond to bone marrow-targeted therapies (e.g., CXCR2 and CSFR inhibitors), while IG2 may benefit from CDK inhibitors and strategies regulating HLA and APM transcription. Overall, SCS provides a new perspective on liver cancer immune landscapes and potential TME-targeted therapies.

### Pancreatic cancer

Pancreatic ductal adenocarcinoma (PDAC) accounts for about 90% of pancreatic cancers and is an emerging malignancy. Its incidence has doubled in the past 20 years and is projected to become the second leading cause of cancer-related death by 2030 [140]. PDAC is characterized by an extremely poor prognosis, with the lowest 5-year survival rate (12%) among all tumors [141]. Tumor heterogeneity is the dominant factor contributing to the poor survival rate of PDAC, leading to diverse cell sets with different sensitivity to therapy. Another vital cause for treatment resistance is the abundance of tumor stromal cells, constituting approximately 90% of PDAC [142].

Analysis of 309 PDAC samples using gene expression and consensus clustering validated the widely accepted basal-like and classical subtypes and identified five subtypes (pure basal like, stroma activated, desmoplastic, pure classical, and immune classical) associated with patient outcomes and therapeutic targets [143]. In the era of SCS, this approach provides a high-resolution view of inter-tumoral and intra-tumoral heterogeneity, enabling comprehensive gene expression profiling in PDAC. A scRNA-seq study of over 40,000 human PDAC cells identified two major malignant ductal subtypes, type 1 and type 2 [52]. (Table 1). In comparison with type 1 cells, type 2 cells exhibit higher expression levels of PDAC markers that are associated with poor prognosis: CEACAM1/5 and KRT19. A subsequent study confirmed the co-existence of classical and basal-like cell clusters within the same PDAC bulk [144]. These clusters were categorized into distinct subtypes with different molecular features: classical-like cells often show *GATA6* amplification and *SMAD4* deletion, whereas basal-like cells frequently harbor *TP53* mutations and *CDKN2A* loss. SCS studies of PDAC metastasis revealed higher proportions of epithelial tumor cells and specific macrophages expressing *CD74*,* FCER1G*, and *MHC I/II* in metastatic tumors compared with primary PDAC [53]. Interestingly, the scRNA-seq focusing on liver metastatic lesions of PDAC identified a ductal cell subpopulation that is functionally correlated with EMT [54]. For CSCs, a comprehensive scRNA-seq study revealed a negative correlation between stemness and the efficacy of immunotherapy in PDAC [145]. In addition, identification of the CSC clusters from PDAC by scRNA-seq allows more accurate validation of CSC-related genes, such as *LY6D* and *MET* [146].

Single-cell profiling also helps identifying stromal heterogeneity in the TME of PDAC. CAFs are mesenchymal stromal cells with complex functions, capable of exerting both positive and negative effects in PDAC [147]. A study conducting scRNA-seq in human and mice PDAC identified seveal subpopulations of CAFs that are also reported in other GI cancers: iCAFs and myCAFs [72] (Table 2). The iCAFs are located distant from the tumor cells and characterized by secreted inflammatory mediators, such as IL-6 and LIF, while notably lacking the αSMA marker. In contrast, myCAFs are adjoining PDAC cells with high levels of αSMA. There are also significant differences in the expression of collagen markers between the two CAF subtypes [148]. Another study identified a third CAF population, termed antigen-presenting CAFs (apCAFs), which express MHC-II and CD74 but lack typical co-stimulatory molecules. It has been demonstrated that apCAFs can directly activate CD4^+^ T cells in *vitro*; however, without proper co-stimulation, this may induce T cell anergy or Treg differentiation, potentially contributing to immune suppression in certain contexts [72]. In summary, the advancement of scRNA-seq technology has enabled precise exploration of the features, localization, and functions of CAFs subtypes in PDAC.

Immunosuppression is a common feature of PDAC microenvironment, containing heterogeneous immune cells and their complex interactions. For example, T cells were found to be 13 clusters, including five CD4^+^ tumor-infiltrating lymphocytes (TILs), seven CD8^+^ TIL cells, and a cycling subset [149]. In these subsets, RBPJ was identified in CXCL13^+^CD4^+^ and CXCL13^+^CD8^+^ T cells, suggesting T-cell dysfunction. The GZMK^+^CD8^+^ T cells expressed EOMES, a molecular indicative T-cell exhaustion, but lacked several other reported exhaustion markers, suggesting a possible intermediate cell state. In addition, CD274 shows an enrichment in a portion of macrophages, which suggests that a specific subpopulation of TAMs may be the target of anti-PD-L1 drugs. In a scRNA-seq analyses of the genetically engineered mouse model of PDAC, a subpopulation of MDSCs exhibit high expressions of S100A8, S100A9, and G0s2, while the overall immune cells display a low myeloid-to-lymphocyte ratio [150].

The status of the diverse cells within PDAC is dynamic, continually evolving, and influenced by therapy interventions. For precursor lesions of PDAC, scRNA-seq uncovered the heterogeneous changes within the epithelium and TME during the evolution of non-invasive atypical hyperplasia to invasive cancer [151]. In the multistep progression of intraductal papillary mucinous neoplasms (IPMNs), the high-grade stage is marked by an increase in iCAFs and elevated expression of the metastasis-associated marker CXCL12. A recent scRNA-seq study of PDAC chemotherapeutic response revealed a heterogeneous mix of basal and classical cancer cell subtypes alongside distinct CAF and TAM populations following treatment. The study also highlighted TIGIT as a key inhibitory checkpoint on CD8^+^ T cells, suggesting potential strategies to overcome chemoresistance in PDAC [152]. Focusing on the tumor microenvironment, single-cell analysis in PDAC identified TGFβ-driven LRRC15⁺ CAFs that surround tumor islets, are absent in normal tissue, and whose high signature correlates with poor anti-PD-L1 response, highlighting a translational target to improve immunotherapy outcomes [153]. Extending the insights into chemoresistance, a scRNA-seq and spatial transcriptomics study of PDAC under neoadjuvant therapy revealed three multicellular communities and enrichment of acinar cells expressing REG1A and REG3A, highlighting microenvironment remodeling [154].

In total, SCS technology uncovered the subtypes of cancer and stromal cells, linked these subtypes to their specific gene expression signatures, and revealed their complex interactions within the TME. By connecting the signatures to predicted or experimentally validated functions, such as immunosuppression, matrix remodeling, or therapy resistance, SCS captures the dynamic transformation of cells during tumor progression. These insights highlight the translational potential of single-cell analyses to guide subtype-specific therapies and advance precision medicine for pancreatic cancer.

### Targeting the synthesizing mechanisms in GI cancers

Single-cell sequencing studies in GI cancers have increasingly uncovered cross-cancer mechanisms and key cellular subsets that drive tumor progression. Among these, SPP1⁺ macrophages have emerged as a particularly important population in gastric cancer, CRC, and HCC [44, 111, 129]. These cells exhibit multiple pro-tumorigenic functions in the TME, including immunosuppression through inhibition of CD8⁺ T cell and NK cell activity and promotion of Treg expansion, facilitation of angiogenesis, enhancement of tumor invasion and metastasis via extracellular matrix remodeling, and modulation of the inflammatory milieu. Collectively, these findings underscore SPP1⁺ macrophages as critical drivers of tumor development in GI cancers. Importantly, the lead compound CANDI460 can down-regulate SPP1 both in *vitro* and in *vivo*, resulting in tumor remissions across multiple murine models, highlighting the therapeutic potential of targeting this macrophage subset [155].

FAP⁺ CAFs have likewise been identified as a key cellular component of the TME in various GI cancers [112, 156]. These fibroblasts interact closely with TAMs to cooperatively promote angiogenesis and facilitate immune evasion, thereby supporting tumor growth and progression. In this context, a first-in-human study of the bispecific fusion protein RO7122290, which costimulates T cells via a 4-1BB ligand and targets FAP, demonstrated both peripheral and intratumoral T cell activation as well as clinical responses in patients with advanced solid tumors, supporting further evaluation in combination with atezolizumab or other immuno-oncology agents [157].

### Limitations of scRNA-seq

Currently, scRNA-seq technology faces numerous challenges and limitations in characterizing the transcriptomic landscape of individual cells and in its clinical applications. First, the transcriptome, that covers the mRNA, only partially reflects the expression of corresponding proteins due to the post-transcriptional regulation, e.g. by microRNAs [158], which limits the interpretation of scRNA-seq results [159]. Consequently, conclusions drawn solely from scRNA-seq may not be entirely accurate. To obtain robust and reliable findings, it is often necessary to validate results using independent external cohorts, integrate scRNA-seq and spatial transcriptomic data with proteomic or metabolomic datasets, and perform experimental verification.

A technical limitation is that scRNA-seq fails to reliably detect low-abundance transcript information and can only detect approximately 10% of transcripts from a single cell, with up to 60% RNA content is lost, which may mask potentially critical biological variants [160, 161]. For example, long non-coding RNAs, which play a key role in epigenetic regulation, are often undetectable by scRNA-seq due to the fact that they are often present in multiple copies [162]. Therefore, improving the sensitivity of scRNA-seq is necessary to provide a full understanding of tumor heterogeneity. Other non-coding RNAs, such as circular RNA or microRNA, are also not addressed by this technology. Other technical limitations include the absence of a gold standard for annotating cell clusters to specific cell types during unsupervised clustering, and the fact that downstream analyses such as trajectory inference methods are still in their infancy, which places greater demands on sample preparation and DNA barcoding methods [163, 164]. Recognising the role of tumor-environment interactions, there is also a need to better delineate the role of local microbial factors in triggering molecular pathways. Last but not least, the quality and outcome of scRNA-seq analysis is strongly dependent on the quality of the primary tissue, which is a significant limitation for several entities, particularly in pancreatic cancer.

Beyond technical limitations, methodological constraints should not be overlooked. Overclustering, which may lead to the identification of spurious subtypes without functional distinction, is a significant concern. Similarly, batch effects, sampling bias, and challenges in integrating datasets across studies are critical issues that researchers must consider. Moreover, due to the heterogeneous composition of tissue samples, comparing cell proportions across samples is challenging, especially when samples are not enriched using specific markers such as CD45 [165].

### Future perspectives

**Fig. 4:**
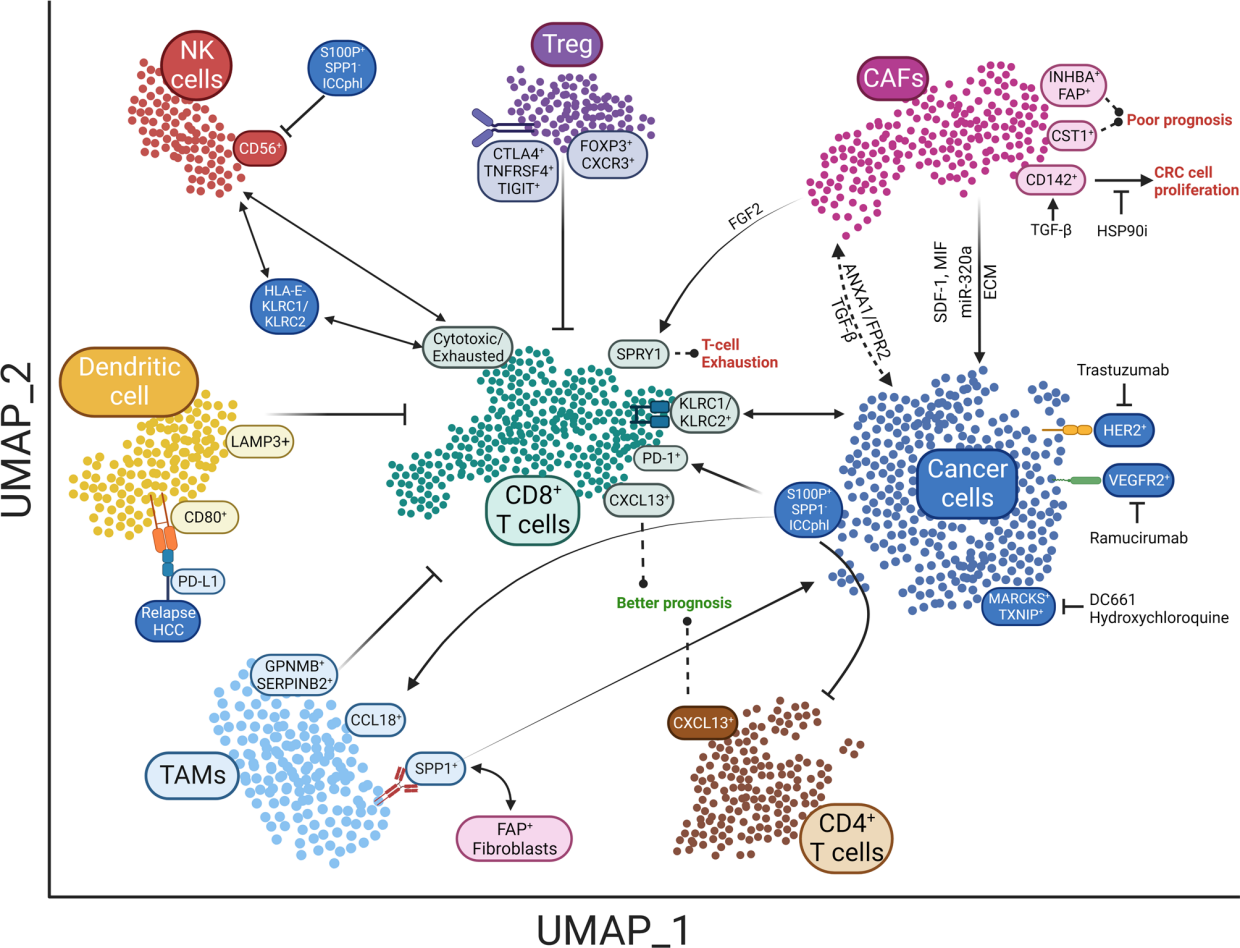
Crosstalk between various types of cells in the TME of GI cancers. SCS revealed complex crosstalk between different cell clusters. Interactions between immune cells, stromal cells, and tumor cells are the most extensively studied ones in the TME. The Figure illustrates the scRNA-seq-based studies of GI cancers in recent years. SCS: Single-cell sequencing; TME: Tumor microenvironment

The rapid development of targeted therapies and immunotherapies has made it possible to individualize the treatment of GI cancers. However, it has also placed higher demands on our understanding of the cellular heterogeneity and plasticity within tumors. Meanwhile, tumor heterogeneity is one of the major challenges of implementing personalized medicine in GI cancers. Currently, numerous SCS-based studies have successfully performed omics sequencing and subtype classification of single cells in the TME of GI cancers, which provides a comprehensive feature landscape for each cell type in the TME and advances our understanding of the dynamics of GI cancers in the process of disease progression and therapeutic response (Fig. 4; Table 1). These findings will provide a favorable pathway to resolve tumor heterogeneity, better define the molecular subtype of the TME, and develop more effective combination therapies and individualized interventions.

In addition to tissue based approaches which are the majority in SCS technologies right now, the high resolution of SCS can also be used to characterize rare cells within the blood. Circulating tumor cells as well as host cells can nowadays be assessed to fully understand the metastatic cascade and (hematogenous) progression in GI tumors through liquid biopsy [166]. Moreover, novel liquid biopsy analytes such as circulating cancer associated fibroblasts [167], dormant polyploid giant cancer cells [168], Extracellular vesicles (EVs) [169], circulating endothelial cells [170] and tumor-educated platelets [171] can be analyzed to better understand the role of the host and the TME in GI cancer patients.

Besides the analysis of novel analytes such as the blood, there is also an increasing interest in combining different techniques and layers of information to enhance the information provided by SCS. For example, pathology imaging techniques such as FISH with scRNA-seq to study single-cell transcriptome profiles at the spatial level, or integrating scRNA-seq with other single-cell omics including genome (like scTrio-seq, G&T-seq, and DR-seq), epigenome (like scM&T-seq for DNA methylation, sci-CAR for chromatin accessibility), and proteome (like CITE-seq and REAP-seq) to provide more comprehensive insights [172, 173]. In addition, some comprehensive databases containing single-cell multi-omics data like scMoresDB are being established [174]. As the number of scRNA-seq datasets increases rapidly, the computational methods for data analysis need constant optimization. Overall, the prospects of SCS technology in the research field of GI cancers are highly promising. Many of the current research findings cannot yet be immediately translated into clinical practice. Furthermore, public understanding of the application of SCS is insufficient, and the currently high testing costs make a satisfactory cost-benefit ratio difficult for SCS in precision medicine. Nevertheless, we remain optimistic that advances in SCS technology will gradually improve our knowledge of tumor biology.

This, in turn, will help to explore novel therapeutic targets and achieve precision treatment of GI cancers in the future.

## Data Availability

No datasets were generated or analysed during the current study.

## Abbreviations

CAF: Cancer-associated fibroblast
CDC27: Cell division cycle 27
CECSC: CRC Subtyping Consortium
CHC: Combined hepatocellular and intrahepatic cholangiocarcinoma
CMS: Consensus molecular subtypes
CRC: Colorectal cancer
CRC-LM: Colorectal cancer liver metastases
CSC: Cancer stem cell
DC: Dendritic cell
EAC: Esophageal adenocarcinoma
ESCC: Esophageal squamous cell carcinoma
FLG: Filaggrin
GI: Gastrointestinal
GO: Gene ontology
GSEA: Gene set enrichment analysis
HCC: Hepatocellular carcinoma
HSC: Hepatic stellate cell
ICC: Intrahepatic cholangiocarcinoma
IPMN: Intraductal papillary mucinous neoplasm
KEGG: Kyoto Encyclopedia of Genes and Genomes
KNN: K-nearest neighbor
m6A: N6-methyladenosine
MDSC: Myeloid-Derived Suppressor Cell
MSI: Microsatellite instability
OS: Overall survival
PBMC: Peripheral blood mononuclear cell
PCA: Principal component analysis
PDAC: Pancreatic ductal adenocarcinoma
dMMR: Mismatch repair-deficient
SCS: Single-cell sequencing
scRNA-seq: Single-cell RNA sequencing
SNV: Single-nucleotide variant
SPRY1: Sprouty RTK signaling antagonist 1
TAM: Tumor-associated macrophage
Treg: Regulatory T cell
TIL-Bs: Tumor-infiltrating B lymphocytes
TI: Trajectory inference
TIL: Tumor-infiltrating lymphocyte
TME: Tumor microenvironment
t-SNE: t-distributed stochastic neighbor embedding
UMAP: Uniform manifold approximation and projection
